# Non-pharmacological Approaches to Managing Attention-Deficit Hyperactivity Disorder in Pediatric Populations: A Scoping Review

**DOI:** 10.7759/cureus.87810

**Published:** 2025-07-13

**Authors:** Nisarg Shah, Harshita Nadella, Shane Williams

**Affiliations:** 1 Cardiology, Rheumatology, Nova Southeastern University Dr. Kiran C. Patel College of Osteopathic Medicine, Davie, USA; 2 Rheumatology, Immunology, Nova Southeastern University Dr. Kiran C. Patel College of Osteopathic Medicine, Davie, USA; 3 Family Medicine, Nova Southeastern University Dr. Kiran C. Patel College of Osteopathic Medicine, Davie, USA

**Keywords:** adhd in school children, attention deficit hyperactivity disorder (adhd), dietary intervention, exercise intervention, non-pharmacological interventions, pediatrics

## Abstract

Attention-deficit hyperactivity disorder (ADHD) is the most common neurocognitive disorder diagnosed in children, with an estimation of six million children aged 3-17 diagnosed with ADHD. Stimulants are the first-line treatment for children with ADHD but may cause adverse effects. This study aims to shed light on non-pharmacological approaches to treating children with ADHD, specifically exercise and dietary modifications. This literature-based review evaluated current research on PubMed focusing on the impact of exercise and dietary modifications as forms of treatment for ADHD. Though studies differ on what symptoms are most improved, exercise was found to be cost-effective and beneficial in ADHD symptom management. Of note, hyperactivity and aggressive behavior improved in children with ADHD after a yoga regimen was implemented in one study. In addition to exercise, the literature review found that a healthy diet consisting of vegetables, fish, and polyunsaturated fatty acids (PUFAs) is the recommended choice for aiding in ADHD symptom management. Omega-3, zinc, and iron supplementations are not recommended due to no documented consistent effect on reducing symptoms.

## Introduction and background

Attention-deficit hyperactivity disorder (ADHD) is a neurodevelopmental disorder. It is one of the most commonly diagnosed neurodevelopmental disorders in children. Children under the age of 18 who have ADHD have outnumbered those with autism by almost four to one [1]. Symptoms usually show up between the ages three and six years and the average age at diagnosis is seven years. In order to diagnose ADHD, the Diagnostic and Statistical Manual of Mental Disorders, fifth edition (DSM-V) criteria require that six or more symptoms of inattention be present in children up to the age of 16 and must also be present in more than two settings, such as home, school, or other activities [1]. Table 1 provides further details. In addition to the details discussed in Table 1, there should be a reduced quality of function in the individual’s life, and the symptoms should not fit the criteria for any other diagnosis.

**Table 1:** Summary of DSM-5 ADHD diagnosis features for children. Five or more symptoms from each category or five symptoms from each of the two categories for a total of 10 or more with at least some symptoms before the age of 12 [1]. DSM-5: Diagnostic and Statistical Manual of Mental Disorders, fifth edition (DSM-V); ADHD: attention-deficit hyperactivity disorder

Category of Symptom	Examples
Inattention symptoms	Fails to pay attention to detail, difficulty maintaining attention, difficulty listening, keeping up with conversation, difficulty with organization, avoids tasks that require prolonged attention, can’t keep track of personal items, easily distracted, forgetful
Hyperactivity and impulsivity symptoms	Fidgety or squirmy behavior, trouble remaining seated for extended periods of time, restless, unable to engage in activities quietly, predominantly on the go, overly talkative, talks over others, cuts off other people in conversation, trouble with patience and waiting for one’s turn

According to the Centers for Disease Control and Prevention (CDC), there are an estimated six million children between the ages of three and 17 years diagnosed with ADHD [1]. Children with ADHD most likely suffer from symptoms such as inactivity, hyperactivity, and impulsivity. Even though ADHD is more common in children, ADHD usually persists throughout a person’s lifetime; approximately two-thirds of children continue to have symptoms throughout adulthood [2]. Even though some researchers have suggested that ADHD is more prevalent in men, more case studies are showing that ADHD is equally prevalent in women but underdiagnosed due to multiple factors with bias being one. 

Around 2.8% of adults around the world have been diagnosed with and are being actively treated for ADHD and these symptoms can cause difficulties in relationships, at home, and at work [2]. However, some studies show that around 6.7% (amounting to 360 million) of adults worldwide are affected by ADHD symptoms but do not get diagnosed and do not seek treatment [2]. It is important to recognize, however, that while symptoms in children may include being unable to sit still, fidgeting, and excessive talking and movement, symptoms in adults may look more like misplacing items, always running late, inability to prioritize tasks, and relationship troubles. 

Although there is no direct cause for ADHD, there are many known and researched risk factors such as low birth weight, being born prematurely, use of alcohol and tobacco during pregnancy, and some brain injuries [3]. It is also strongly hypothesized that children with ADHD have a frontal lobe that might mature more slowly in comparison to those children without [3]. As one can see from Table 1, even though there are many different symptoms of ADHD, they can broadly be categorized into two groups: inattentive and hyperactive-impulsive. Inattentive ADHD can look like difficulty completing tasks and being easily distracted day to day. This subtype of ADHD is often also referred to as ADHD, predominately inattentive presentation (formerly referred to as "ADD") [4]. Predominately hyperactive-impulsive presentation ADHD can present in people who tend to talk a lot, are interruptive, restless, and speak at inappropriate times [4]. There is also a third subtype - the combined type - in which individuals exhibit symptoms from both categories [4].

Based on the updated guidelines by the American Academy of Pediatrics, preschool-aged children (four to six years old) with ADHD should be managed initially with parent training in behavior management (PTBM), with methylphenidate serving as an adjunct if there is a continued disturbance in function despite the behavioral interventions [5]. Methylphenidate is a stimulant that increases norepinephrine and dopamine levels in the brain, which allows patients to focus. For elementary and middle school-aged children (six to 12 years old) with ADHD, the first-line treatment consists of US Food and Drug Administration (FDA)-approved medications for ADHD along with PTBM and behavioral classroom interventions. Regarding FDA-approved medications, stimulants like methylphenidate have strong data supporting their use, while other medications, such as atomoxetine, extended-release guanfacine, and extended-release clonidine, have sufficient, but not strong, data to support their use [5]. For adolescents (12-18 years old) with ADHD, first-line management includes US FDA-approved medications for ADHD. Educational interventions are also a necessary aspect of ADHD management in this population such as tailored classroom mentoring and individualized educational plans [5].

With stimulants being the first-line medication choice for ADHD, the adverse effects of stimulants should not be overlooked. Common stimulant side effects include headache, appetite suppression, abdominal pain, and delay of sleep onset. There have also been cases of tics exacerbation and stimulant-induced mood dysregulation [6]. Furthermore, there is also a slight risk of growth rate reduction in both height and weight due to stimulant use [6]. Stimulants like methylphenidate can also cause withdrawal symptoms if abruptly discontinued after prolonged use [7].

In light of the potential adverse effects associated with stimulant medications, this paper aims to explore non-pharmacological interventions for managing ADHD in children. In recent times, interventions such as psychosocial treatments, behavioral management, exercise, mind-body interventions like meditation and yoga, and dietary modifications have been explored [8]. Exercising and dietary modifications have been encouraged due to their many benefits on health, ranging from lowering the risk of cardiovascular disease to promoting bone and gut health [9,10]. Exercise regimens, consisting of method, duration, and type, are explored in this paper. Dietary modifications, such as supplementation diets, elimination diets, and the validity of omega-3 effects on ADHD, are also accentuated.

## Review

Methods

This literature-based review evaluated current research focusing on the impact of exercise and dietary modifications as forms of treatment for ADHD. PubMed served as the primary database for article retrieval due to access challenges. Inclusion criteria included research published in the United States, articles had to be published from 2014 onwards, and the subjects included in the studies had to be younger than or equal to 18 years of age. Two searches were conducted on March 23, 2024: One for the influence of exercise on ADHD and another for the effects of dietary modifications on ADHD. Articles were first screened to ensure they were published in the English language.

**Figure 1:**
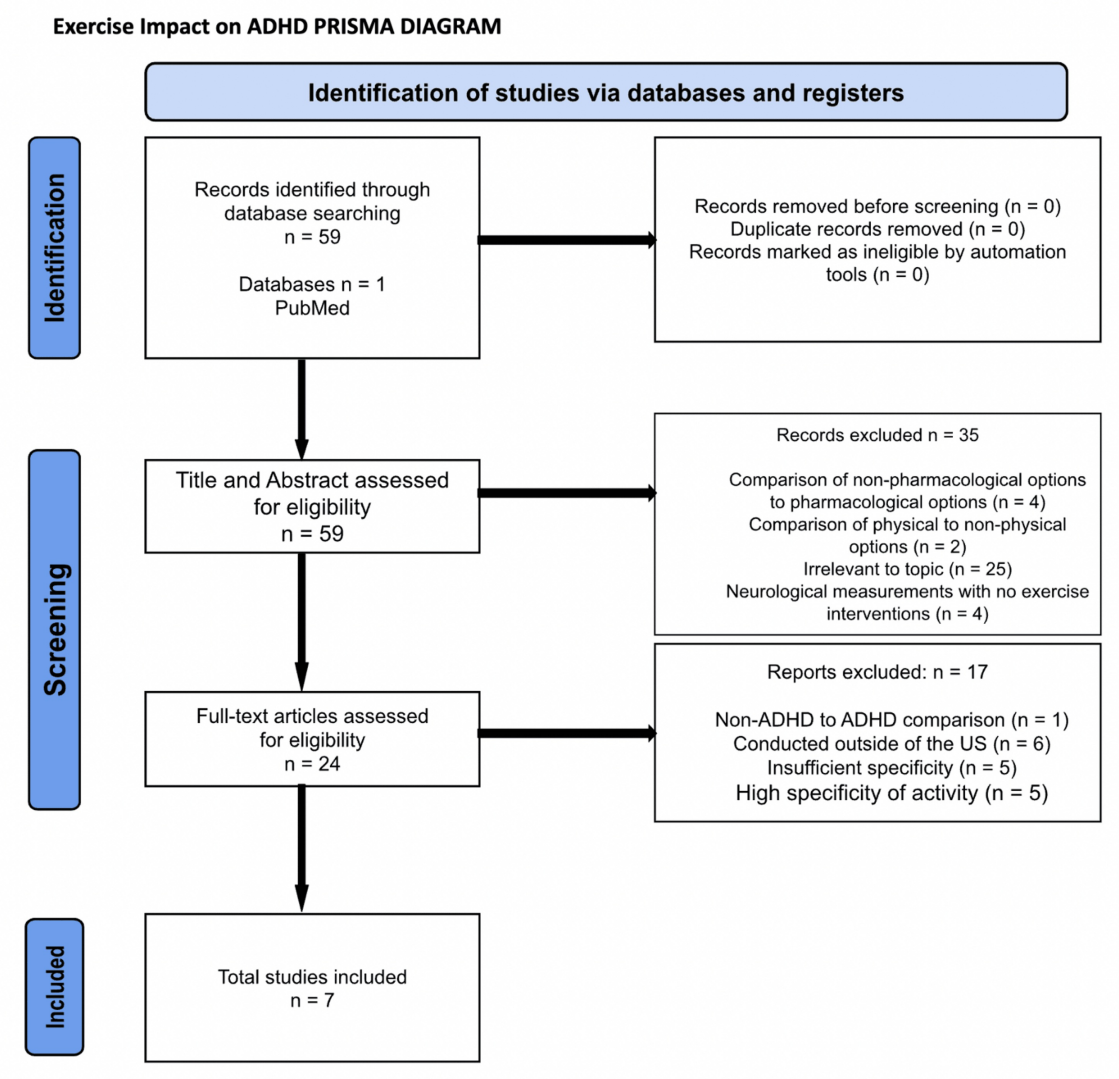
Exercise Impact on ADHD: Preferred Reporting Items for Systematic Reviews and Meta-Analyses (PRISMA) Diagram ADHD: Attention-deficit hyperactivity disorder.

A total of 59 articles were identified and screened for the effect of various parameters regarding exercise on the management of ADHD. The search term used was “physical activity as a treatment for ADHD in children”. These articles were then screened to identify those that focused on the method of exercise, mode of exercise, duration, and impact on particular symptoms of ADHD. This yielded an inclusion of 10 articles. This process can be visualized in the Preferred Reporting Items for Systematic Reviews and Meta-Analyses (PRISMA) diagram in Figure 1.

**Figure 2:**
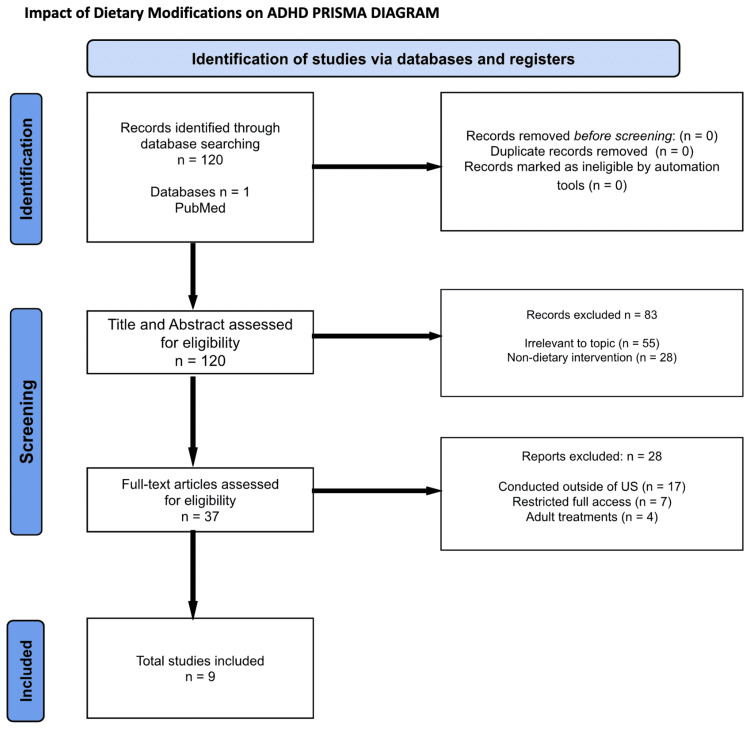
Impact of Dietary Modifications on ADHD: Preferred Reporting Items for Systematic Reviews and Meta-Analyses (PRISMA) Diagram ADHD: Attention-deficit hyperactivity disorder.

A separate search was conducted to assess the impact of dietary modifications, in particular, on the management of ADHD. A total of 120 articles were identified and screened by the inclusion criteria, as well as their relevance to this topic. The search term used for this section was "food" and "treatment" and "ADHD" and "children". These articles were further screened to identify the particular ones that directly examined the effects of certain diets, whether it be specific supplements or eliminations, on ADHD symptoms. A final selection of nine articles was included. The PRISMA diagram in Figure 2 can be utilized for further visualization.

Results

After our final number of sources were chosen according to the criteria listed above, articles were organized according to the type of intervention utilized to address ADHD symptoms, specifically exercise-related interventions versus dietary interventions. All articles were written in English and published as US-based literature. Out of the total of 16 articles considered, three references are review articles, two are experimental studies, three are meta-analyses, one is a meta-analysis and a systematic review, six are systematic reviews, and one is a narrative review.

The discussion began with a broader overview to highlight the clinical significance of ADHD and the low levels of physical activity observed in the pediatric population. Then using sources [11-13], this paper lays the biological foundation for how exercises specifically can help brain function for those suffering from ADHD (Table 2). In addition, with sources [14-17], the paper attempted to elucidate what particular ADHD symptoms were helped by a particular exercise (Table 2). Executive function was linked with open-skill sports such as basketball, tennis, and soccer while attention and memory was improved when the pediatric population took part in long-term consistent aerobic exercises such as those that focus on cardio. Working memory was closely associated with closed skills such as gymnastics and archery and hyperactivity was highly improved with exercises such as yoga.

**Table 2:** Summary of Citations for Physical Interventions ADHD: Attention-deficit hyperactivity disorder, NIH: National Institutes of Health.

Reference Number	Author	Year of Publication	Study of Title	Journal Name	Study Type	Key Findings
[11]	Collins	2016	Exercise releases brain-healthy protein	NIH Director's blog	Review article	Highlights the role of cathepsin B, a protein secreted during exercise, in enhancing memory via hippocampal neurogenesis - key for ADHD-related memory deficits. Suggests molecular pathways linking physical activity to improved cognitive function
[12]	Chan et al.	2022	Effects of physical exercise on children with ADHD	Biomedical Journal	Experimental study	Demonstrates that moderate-intensity exercise significantly improves attention and executive function in children with ADHD, supporting its use as an adjunct treatment targeting core cognitive deficits.
[13]	Christiansen et al.	2019	Effects of exercise on cognitive performance in children and adolescents with ADHD: potential mechanisms and evidence-based recommendations	Journal of Clinical Medicine	Review article	Proposes that exercise enhances cognition through increased neurotransmitter levels (dopamine, norepinephrine) and white matter integrity - particularly in regions tied to attention and working memory, supporting its relevance for ADHD and brain development.
[14]	Sun et al.	2022	Effects of physical exercise on attention deficit and other major symptoms in children with ADHD: a meta-analysis	Psychiatry Research	Meta-analysis	Confirms that aerobic exercise significantly improves inattention and memory in children with ADHD. Chronic activity produced stronger outcomes than acute bouts, reinforcing exercise’s potential as a long-term intervention.
[15]	Den Heijer et al.	2017	Sweat it out? The effects of physical exercise on cognition and behavior in children and adults with ADHD: a systemic literature review	Journal of Neural Transmission	Systematic review	Identifies acute aerobic activity (e.g., running, jumping) as improving executive function (planning, inhibition, attention), while chronic activity improves memory. Aligns with this paper’s focus on exercise modality and symptom-specific impacts.
[16]	Zhu et al.	2023	Comparative effectiveness of various physical exercise interventions on executive functions and related symptoms in children and adolescents with attention deficit hyperactivity disorder: a systematic review and network meta-analysis	Frontiers of Public Health	Systematic review and network meta-analysis	Compares open-skill (e.g., sports) vs. closed-skill (e.g., running) exercises. Open-skill exercises showed greater improvements in inhibitory control, while closed-skill improved working memory - emphasizing the role of task complexity in ADHD outcomes.
[17]	Zang	2019	Impact of physical exercise on children with ADHD: meta-analysis	Medicine	Meta-analysis	Shows moderate-to-large improvements in inattention and memory with physical activity while hyperactivity/impulsivity improved.

Aside from the exercise interventions, the remaining articles were categorized under dietary interventions. Nine articles selected addressed the role of diet and dietary interventions on affecting ADHD symptoms. Two of these articles, sources [18] and [19], discussed the association of unhealthy dietary patterns with an increased ADHD prevalence. Four articles, sources [20-23], highlighted the effects of dietary changes and supplementations on ADHD symptoms. Four articles, sources [24-27], delved into omega-3 fatty acids in particular and the effects of incorporating them into the diet on ADHD symptoms. Of the nine articles selected for this section of the paper, one was a meta-analysis, one was a narrative review, six were systematic reviews, one was a review article, and one was an experimental study. Table 3 provides details of the studies used for this section of the paper.

**Table 3:** Summary of Citations for Dietary Interventions ADHD: Attention-deficit hyperactivity disorder, FFD: few foods diet, PUFA: poly-unsaturated fatty acid, AFC: artificial food coloring, EFA: essential fatty acids, RED: restricted elimination diets, AFCE: artificial food coloring elimination

Reference Number	Author	Year of Publication	Study of Title	Journal Name/Publisher	Study Type	Key Findings
[18]	Zhu et al.	2023	Risk of overweight and obesity in children and adolescents with attention-deficit/hyperactivity disorder: a systematic review and meta-analysis	Childhood Obesity	Meta-analysis	Children with ADHD have a significantly higher likelihood of concurrent obesity.
[19]	Pinto et al.	2022	Eating patterns and dietary Interventions in ADHD: a narrative review	Nutrients	Narrative review	Unhealthy diets were positively associated with ADHD symptoms, while healthy patterns were negatively associated; Vitamin D and magnesium seemed to improve symptoms when there was a vitamin D insufficiency present
[20]	Pelsser et al.	2017	Diet and ADHD, reviewing the evidence: a systematic review of meta-analyses of double-blind placebo-controlled trials evaluating the efficacy of diet interventions on the behavior of children with ADHD	PLoS One	Systematic review	FFD intervention had a significant effect on ADHD symptoms, whereas PUFA supplementations and AFC interventions did not significantly affect ADHD symptoms
[21]	Hurt and Arnold	2014	An integrated dietary/nutritional approach to ADHD	Child and Adolescent Psychiatric Clinics of North America	Systematic review	Highlighted several key points, including EFA supplementation, zinc adjunctive therapy, and certain Chinese herbs showing improvement in ADHD symptoms; several of these studies were conducted to show benefit as adjunctive therapies with methylphenidate
[22]	Stevenson et al.	2014	Research review: the role of diet in the treatment of attention-deficit/hyperactivity disorder - an appraisal of the evidence on efficacy and recommendations on the design of future studies	Journal of Child Psychology and Psychiatry, and Allied Disciplines	Systematic review	REDs showed some benefit in treating ADHD symptoms; AFCE has also shown potential in treating ADHD symptoms, but this study was unable to determine a proper effect size
[23]	Hariri and Azadbakht	2015	Magnesium, iron, and zinc supplementation for the treatment of attention deficit hyperactivity disorder: a systematic review on the recent literature	International Journal of Preventive Medicine	Systematic review	Not enough significant evidence for the overall recommendation of iron, magnesium, and zinc for children with ADHD
[24]	Krupa et al.	2023	Omega-3 fatty acids	StatPearls	Review article	Benefits of omega-3 fatty acids in the context of ADHD are being explored, as it is a well-known dietary supplement
[25]	Kemper et al.	2018	Attention deficit hyperactivity disorder: diagnosis and treatment in children and adolescents	Agency for Healthcare Research and Quality	Systematic review	Adding psychosocial/behavioral interventions to psychostimulants is more effective than psychosocial/behavioral interventions alone; Omega-3/6 supplementation does not appear to improve ADHD outcomes
[26]	Gow et al.	2015	Current evidence and future directions for research with omega-3 fatty acids and attention deficit hyperactivity disorder	Current Opinion in Clinical Nutrition and Metabolic Care	Systematic review	While omega-3 supplementation benefited certain patients, it showed no benefit for other patients

Discussion: impact of physical exercise

Observing that ADHD is one of the most common neurodevelopmental disorders that children are diagnosed with and obesity is also a rising concern in this country, looking at exercise as a possible solution for both of these chronic conditions is vital.

Although it might be well established that exercise is beneficial for one’s health, there has been new research shedding light on specific neurochemical pathways as to why exercise is beneficial for those with ADHD. First, physical exercise is linked with improved memory. When one starts to exercise, the skeletal muscle cells starts to secrete a protein called cathepsin B [11]. Researchers not only found that as levels of this protein increase, memory also increases but also that memory is diminished in those unable to produce cathepsin B [11]. This highlights the potential of exercise to boost cognitive function and memory in individuals with ADHD, particularly through molecular mechanisms that are linked to neurogenesis and memory enhancement. Second, any form of physical activity causes increased blood flow to the brain, which in turn promotes cell growth, particularly in the hippocampus. This is the part of the brain that is linked with memory and concentration. This increased blood flow leads to neuroplasticity in nerve cells and synaptic connection, which not only promotes attention and concentration but also strengthens those existing connections [12].

Researchers also believe that exercise acts as an “endogenous stimulus to trigger a cascade of molecular neuroplastic processes” to promote higher levels of cognitive function. This has already been in use to prevent atrophy of brain volume seen in aging or neurocognitive diseases such as Alzheimer's but new research is now showing positive results in children as well. Children who participate in higher forms of exercise have demonstrated “lower grey matter thickness”, which is associated with increased memory and cognitive reasoning. Furthermore, there are other exercise-induced structural adaptations in children and adolescents such as “white matter integrity in frontotemporal bundles”, which are activated in higher-order thinking and found in higher numbers in those without ADHD in comparison with those with ADHD. These changes are more commonly observed in individuals without ADHD, suggesting that exercise may help close the cognitive gap between children with and without ADHD [13].

Lastly, the increased brain blood flow increases the number of neurotransmitters such as serotonin, dopamine, and brain-derived neurotrophic factor (BDNF) [12]. In ADHD, there is “abnormal monoaminergic neurotransmission”, which negatively affects attention and reward processing [13]. With exercise of moderate intensity, there was an increase in “catecholamines with longer lasting increments of dopamine”; the increase in neurotransmitters and increased duration of said neurotransmitters in synapses are associated with not only better concentration but also better mood and emotional stability [13]. In ADHD, abnormal neurotransmission often impairs attention and reward processing. However, moderate exercise has been shown to increase catecholamines, especially dopamine, which can enhance mood and concentration [13]. This neurochemical cascade is critical in addressing the cognitive and emotional symptoms often associated with ADHD.

Several studies have examined the specific effects of different types of physical activity on ADHD symptoms. Specifically, acute aerobic exercises such as running or jumping have been shown to improve executive function, including selective attention, task planning, and information processing [14]. On the other hand, chronic aerobic exercises have demonstrated improvements in attention and memory skills, which are commonly deficient in children with ADHD [14]. Additionally, activities requiring higher levels of executive control, such as team sports, have shown more significant improvements in inhibitory control in children with ADHD, as compared to closed-skill exercises like running or cycling [15]. Overall, exercise leads to improved blood flow and aerobic oxygenation to certain parts of the brain such as the hippocampus and fronto-temporal areas. There is an increased number of memory-improving proteins such as cathepsin B and neurotransmitters such as dopamine leading to notable improvements in concentration and moods. Several studies have not only explored why exercise has a positive effect on those with ADHD but also which specific symptoms of ADHD have improved and what mode of exercise addresses those the best. For example, Sun et al. illustrate how exercise can have significant improvement on “executive function and motor skills” [14]. Specifically, this study focuses on how only moderate to intense physical activity can do this. They focus on how moderate to intense physical activity can help with “inattention symptoms” of ADHD [14].

Den Heijer et al. specifically compare what type of exercise addresses certain ADHD symptoms. It was concluded that acute cardio exercises such as “running and jumping” resulted in improved executive functioning such as “selective attention/information processing, inhibiting and planning” [15]. This executive function was tested by comparing results in various tasks that demanded cognitive engagement, anticipation, task demands, and strategic thinking. Long-term and consistent cardio exercises, on the other hand, were linked with improved attention and memory skills, which are also noted to be decreased in children with ADHD [15].

Furthermore, Zhu et al. aim to focus their work more on analyzing the aftermaths of comparing open skill to closed skilled activities. While open skills are performed more in dynamic and changing environments, closed skills tend to take place in more stable and static environments. Comparatively, open skill activities demand a higher level of executive function and as a result, research illustrates that those activities were more beneficial for improving “executive function and specifically inhibitory control” in children with ADHD. Sports are a perfect example of open-skilled activities; in this specific study, children with ADHD who played football had improved inhibitory control. As discussed above, exercise leads to enhanced function of the brain and this study demonstrated that long-term participation caused the neural network to be more closely connected, thus stimulating the inhibitory network functioning. It is hypothesized that long-term participation helps children form a directional action pattern, which allows them to not only standardize their behavior but also become less impulsive. On the other hand, closed-skilled activities such as running or cycling showed significant improvement in working memory in children with ADHD, especially those with long-term training. Specifically, aerobic closed-skilled activities were found to lower dopamine and help with concentration and memory [16]. 

While multiple studies highlight exercise as a cost-effective intervention for ADHD, they differ in identifying which specific symptoms show the greatest improvement. Although there is ample evidence to show that executive function, memory, and inattention have improved with exercise, there has not been sufficient evidence that shows statistically significant improvement in either hyperactivity symptoms or aggressive behavior. It is important to note that a study conducted in 2019 showed that “hyperactive and impulsive symptoms” were improved with physical exercise, but those results were not statistically significant with a p-value of 0.15 [17]. Gunnell et al., however, does show that hyperactivity and aggressive behavior have improved in children with ADHD after 60 minutes of yoga performed once a week for 20 weeks [14]. Though yoga does not fall under the domain of moderate to intense physical activity, this can be an approach of significance for those with predominantly hyperactive symptoms.

Discussion: dietary interventions

Together with exercise benefiting both ADHD and obesity, dietary modifications have been explored to treat obesity as well as ADHD. According to a literature review conducted by Zhu et al., ADHD was associated with a significant risk of having concurrent obesity in children, with an odds ratio (OR) of 1.56 (95% confidence interval (CI), 1.32-1.85) [18]. This association proposes a potential connection between increased body mass index (BMI) and the levels of various neurotransmitters in the brain that produce ADHD symptoms.

Furthermore, healthy diets have been negatively associated with ADHD symptoms, while unhealthy diets have been positively associated with ADHD symptoms. This review outlined several individual studies that investigated the effects of diets, specific foods and supplements on ADHD symptoms, and it found several associations. One review found that a healthy eating pattern consisting of fruits, vegetables, fish, increased polyunsaturated fatty acids (PUFAs), magnesium, and zinc decreased the risk of ADHD by 37 percent (OR: 0.63; 95% CI: 0.41-0.96), whereas a Western-type diet (a diet with increased red and processed meats, refined cereal grains, soft drinks, and hydrogenated fats) increased the risk of ADHD by 92 percent (OR: 1.92; 95% CI: 1.13-3.26) and a junk-food-based diet (a diet with increased processed foods, artificial food coloring, and sugar) increased the risk of ADHD by 51 percent (OR: 1.51; 95% CI: 1.06-2.16). However, studies discussed in this review showed no significant benefit of PUFA, omega-3, and omega-6 supplementations on ADHD symptoms. Vitamin D and magnesium supplementation was found in two studies to show improvement of ADHD symptoms; however, one study concluded that vitamin D supplementation was only beneficial in children with ADHD who also had a concurrent vitamin D deficiency; the other study was designed to add vitamin D and magnesium supplementation to patients taking methylphenidate, so benefits of vitamin D and magnesium supplementation without medications has yet to be shown. Zinc and iron were also investigated for their potential benefits in improving ADHD symptoms. One study showed that zinc and iron supplementation for six to 10 weeks showed improvements, but a systematic review later revealed that after adjusting for baseline zinc levels, zinc supplementation did not show benefit in ADHD. Another review comparing unspecified “healthy” and “unhealthy” diets found that the healthy diet had a protective effect while the unhealthy diet increased the risk of ADHD by 41 percent; the authors of this review also found that children with ADHD had a harder time adhering to a healthy diet compared to children without ADHD, which also highlights another pathway for how various dietary patterns can be associated with ADHD. Probiotic supplementations were also explored to show benefit in ADHD symptoms; several studies showed the supplementation of *Lactobacillus rhamnosus* for 12 weeks improving ADHD symptoms based on self-reports; however, parent and teacher reports showed no difference. Although the benefits of probiotics were shown, not enough research has been done to definitively incorporate probiotic supplementation into the management of ADHD symptoms. Two studies looked into the effects of elimination diets on ADHD symptoms; the TRACE study is ongoing, whereas the INCA study investigated the effects of certain food eliminations on behaviors. An analysis conducted on the INCA study as well as the BRAIN study showed an association between a few-foods diet (FFD) and a decrease in gastrointestinal complaints, eczema, and sleep problems. However, no association was determined between the decrease in these complaints and a decrease in ADHD symptoms. The analysis of these studies showed associations with certain aspects of diets and supplements and effects on ADHD symptoms, but more randomized controlled trials must be conducted before a direct link can be established. Additionally, confounding factors must also be accounted for, as ADHD may be linked with other behavioral and metabolic effects that may indirectly affect dietary intervention outcomes [19].

Dietary modifications, as well as various types of diet supplements, have received attention regarding managing ADHD symptoms, especially for a population like pediatrics where medications are reluctantly started. A literature review consisting of six meta-analyses of studies, including two analyses of artificial food coloring (AFC) eliminations, two analyses consisting of FFD, and two analyses of PUFA supplementations, demonstrated that a FFD intervention had a significant effect on ADHD symptoms, whereas PUFA supplementations and AFC interventions did not significantly affect ADHD symptoms [20]. This study used effect sizes (ESs) as opposed to the traditional p-values to more accurately represent study significance without being significantly affected by sample sizes, since most studies including children tend to have smaller sample sizes. The ESs for AFC elimination and PUFA supplementation were small, while the ES for FFD intervention was large, which showed that using AFC elimination and PUFA supplementations as treatments for ADHD symptoms did not result in a significant improvement, whereas FFD intervention did [20]. Additionally, another literature review compiled the findings from three studies that each looked at the effects of an individual intervention on ADHD symptoms: one study explored a restriction and elimination diet (RED), the second study investigated supplementations of essential fatty acids (EFAs), and the third study researched effects of Chinese herbal medicine [21]. This review highlighted several key points from each study, including EFA supplementation, zinc adjunctive therapy, and certain Chinese herbs, such as duodonging and Ningdong, showing improvement in ADHD symptoms [21]. However, an important factor to note is that several of these studies were conducted to show benefit as adjunctive therapies with methylphenidate treatment as opposed to monotherapies. Additionally, positive benefits were only shown in one or two studies within each of the three primary studies included in the literature review, so replication of these results, in addition to exploring these interventions’ effects when used as monotherapy, are needed before they can be recommended to pediatric patients.

Furthermore, a literature review conducted by Stevenson et al. compared meta-analyses of studies that analyzed the effects of restricted elimination diets (REDs), artificial food coloring elimination (AFCE), and supplementations with free fatty acids (SFFA) on treating ADHD symptoms [22]. REDs showed some benefit in treating ADHD symptoms; however, larger studies that blind the researcher while also comparing its benefit to a control have to be conducted before it can confidently be recommended to children. Nutritional deficiencies are also associated with REDs, so tight monitoring is also needed for patients that are following elimination diets. AFCE has also shown potential in treating ADHD symptoms, but this study was unable to determine a proper ES for AFCE. Combining the findings from this study with the previous review mentioned in this paper conducted by Pelsser et al., the ESs for AFCE were 0.44, 0.21, 0.08, and 0.11 for general scores, parent rating, teacher ratings, and observer ratings, respectively [20]. This shows that AFCE shows potential in mediating ADHD symptoms, but larger studies need to be conducted prior to standardizing AFCE recommendations in children with ADHD. A literature review done in 2015 also investigated the effect of iron, magnesium, and zinc supplementations on ADHD symptoms [23]. This study concluded that there was not enough significant evidence for the overall recommendation of iron, magnesium, and zinc for children with ADHD. Based on the articles analyzed for this review, a few supplementations and diets have been shown to improve ADHD symptoms and show promise, but further studies need to be conducted with consistent results before these interventions can be confidently recommended to children.

Omega-3 fatty acid supplements are currently approved for improving cardiovascular health benefits like treating hypertriglyceridemia [24]. The benefits of omega-3 fatty acids in the context of ADHD are being explored as it is a well-known dietary supplement. A systematic review conducted by Kemper et al. revealed that while child or parent training improved ADHD symptoms (strength of evidence (SOE)=moderate), omega-3 and omega-6 fatty acid supplementation did not improve ADHD symptoms (SOE=moderate); with the strength of evidence being moderate, evidence to support benefit of omega-3 is limited [25]. An additional systematic review conducted by Gow and colleagues found mixed results regarding the impact of omega-3 supplementation on ADHD symptom improvement in children [26]. This study found that while omega-3 supplementation benefited certain patients, it showed no benefit for other patients; further studies will need to be performed to distinguish which population subset benefits from the supplementation [26]. The authors of this study also concluded that it would be a reasonable expectation for patients to use omega-3 supplementation as an adjunct to pharmacologic treatments for ADHD [26]. The adverse effect profile as well as the true quantitative benefit should be investigated further before recommending omega-3 for all patients with ADHD. In 2017, a 12-week pilot study was conducted to observe the effects of omega-3 supplementation in adjunct with medications for ADHD [27]. Deficits in emotional self-regulation (DESR), which include low frustration tolerance and quickness to anger, along with changes in ADHD symptoms, were measured in this study. The omega-3 supplementation was well-tolerated by the patients, but it was shown to only improve the DESR and not the ADHD symptoms [27]. This seems to be consistent with the other studies included in this review. Given the variability in study outcomes, omega-3 supplementation may hold promise for specific subgroups of children with ADHD, but current evidence does not support its routine use in all patients.

This review delved into the impacts of various dietary supplements and dietary modifications on ADHD symptoms with the hopes of finding an intervention that had a significant impact on the symptomatic management of ADHD. Based on the analysis, a healthy diet consisting of vegetables, fish, and PUFAs is the recommended choice for aiding in ADHD symptom management. Omega-3, zinc, and iron supplementations are not recommended since they have no documented consistent benefit in reducing symptoms. Large-scale studies will have to be conducted regarding the various diets and supplements reviewed in this study before they can be recommended properly to pediatric patients with ADHD. Furthermore, randomized controlled trials (RCTs) would offer many benefits to answer the gap recognized in this research.

Limitations

There are several key limitations to this review that should also be highlighted. Firstly, there was only one database used, PubMed, due to constraints in accessibility, as this review was primarily composed during the authors' tenure as medical students. This may have led to some relevant studies being missed. The heterogeneity of study designs used in this paper such as variation on population size and duration of intervention can prove to be challenging to generalize the findings to the larger pediatric population. Secondly, many studies evaluate short-term improvement in executive function or other ADHD symptoms, but long-term data is often lacking. This can be an area of further improvement to follow pediatric patients to adulthood to measure if these interventions have long-term benefits as well if exercise and the set diet persisted. Furthermore, some studies relied on parent or teacher questionnaires, or even child self-reports, which may introduce reporting bias. Finally, inconsistent results regarding several dietary interventions require more stringent and encompassing studies to show consistent benefits before these interventions can be recommended to the pediatric patients with ADHD.

## Conclusions

Both physical exercise and dietary interventions hold promising potential as complementary approaches for managing ADHD symptoms in children, while also addressing the concurrent concern of childhood obesity. Exercise has been shown to enhance cognitive functions such as attention, memory, and executive functioning through neurochemical and structural brain changes, including increased dopamine levels and improved hippocampal function. Different types and intensities of exercise - especially open-skilled and aerobic activities - have shown varying benefits. It is important to note, however, that even though there is some evidence for improvement in hyperactivity and impulsivity, it still remains limited and inconsistent. Similarly, dietary modifications, including adherence to a healthy diet rich in fruits, vegetables, and essential nutrients, have demonstrated some association with reduced ADHD symptoms, while Western and processed diets may actually, in turn, exacerbate them. However, despite some positive findings, many supplements, such as omega-3 fatty acids, zinc, and iron, have not consistently shown benefits across studies, especially when used individually. Ultimately, while both lifestyle interventions show potential, more rigorous, large-scale randomized controlled trials are needed to validate their efficacy and guide evidence-based recommendations. Integrating these strategies into treatment plans as an adjunct may provide a more holistic approach to managing ADHD in children, particularly when pharmacologic therapy is limited or not preferred. Various evidence suggests that integrated treatment plans - which include both medication and behavioral or lifestyle strategies - may be more effective than relying on a single approach. However, integrating both exercises and a healthier diet is becoming of more importance in this day and age due to rising rates of obesity and ADHD as well; but whether those are linked correlations or not demands further research.
